# Gallium-Based Liquid Metal Materials for Antimicrobial Applications

**DOI:** 10.3390/bioengineering9090416

**Published:** 2022-08-25

**Authors:** Chun-Chun Qu, Yu-Tong Liang, Xi-Qing Wang, Shang Gao, Zhi-Zhu He, Xu-Yang Sun

**Affiliations:** 1 College of Engineering, China Agricultural University, Beijing 100083, China; 2 School of Engineering Medicine, Beihang University, Beijing 100191, China; 3 Center for Crop Functional Genomics and Molecular Breeding, China Agricultural University, Beijing 100083, China; 4 Hainan Institute of China Agricultural University, China Agricultural University, Sanya 572000, China

**Keywords:** liquid metal-based materials, gallium, antibacterial mechanism, antibacterial application, antibacterial agents

## Abstract

The hazards caused by drug-resistant bacteria are rocketing along with the indiscriminate use of antibiotics. The development of new non-antibiotic antibacterial drugs is urgent. The excellent biocompatibility and diverse multifunctionalities of liquid metal have stimulated the studies of antibacterial application. Several gallium-based antimicrobial agents have been developed based on the mechanism that gallium (a type of liquid metal) ions disorder the normal metabolism of iron ions. Other emerging strategies, such as physical sterilization by directly using LM microparticles to destroy the biofilm of bacteria or thermal destruction via infrared laser irradiation, are gaining increasing attention. Different from traditional antibacterial agents of gallium compounds, the pronounced property of gallium-based liquid metal materials would bring innovation to the antibacterial field. Here, LM-based antimicrobial mechanisms, including iron metabolism disorder, production of reactive oxygen species, thermal injury, and mechanical destruction, are highlighted. Antimicrobial applications of LM-based materials are summarized and divided into five categories, including liquid metal motors, antibacterial fabrics, magnetic field-responsive microparticles, liquid metal films, and liquid metal polymer composites. In addition, future opportunities and challenges towards the development and application of LM-based antimicrobial materials are presented.

## 1. Introduction

Antibiotic abuse has posed great challenge to bacterial infections, which are serious threats to human health and the medical healthcare system [1]. The emergence of drug-resistant bacteria greatly impairs the effect of traditional antibiotic drugs. Until now, the development of novel antibiotic drugs cannot catch up with the speed of bacterial drug resistance [2]. It is also reported that the economic cost of deaths due to bacterial infections could be as high as USD 81 trillion by 2050 [3], which makes the development of non-antibiotic drugs an urgent need.

Liquid metal (LM) refers to an emerging metallic material with high fluidity at room temperature [4]. In recent years, it has attracted considerable attention from researchers in various fields, attributed to its combined metallic properties and flexibility as a fluidic material as well as its excellent biocompatible property [5]. The applications of LM are mainly focused on wearable electronics [6,7,8], microfluidics [9,10], robotics [11], and thermal conductor materials [12], while recent research has suggested that LM also exhibits biological activities in biomedical therapeutics [13,14,15,16], primarily including tumor hyperthermia, drug delivery, biological imaging, and antibacterial behavior [17,18,19,20,21].

The previous standpoint has demonstrated that the antimicrobial mechanism of gallium (Ga)-based agents was attributed to the similar size and the deceptive behavior of the Ga ion (Ga^3+^) as a metal ion and a competitor of the Fe ion (Fe^3+^) [22], thus interfering with iron (Fe) homeostasis in biological systems and inhibiting the proliferation of bacteria. This point of view has been supported by a large amount of scientific research [23,24,25], and various Ga-based chemosynthetic drugs have been prescribed [26] and approved for use in clinical medicine [27,28]. In addition to the previous use of Ga^3+^ and related compounds as a “Trojan horse” to disrupt Fe metabolism for antimicrobial agents, new forms of Ga-based materials were also developed for antimicrobial applications, and new antibacterial mechanisms were, thus, revealed. For example, it has been revealed that Ga micro/nanoparticles can physically destruct the structure of bacteria and cause irreversible damage to the bacterial cells, which is derived from the transformation and formation of sharp edges in response to external magnetic field or laser irradiation [29]. Meanwhile, the invasion of Ga nanoparticles into cells that causes a surge in reactive oxygen species (ROS) concentration could induce apoptosis as well [29,30,31].

**Figure 1:**
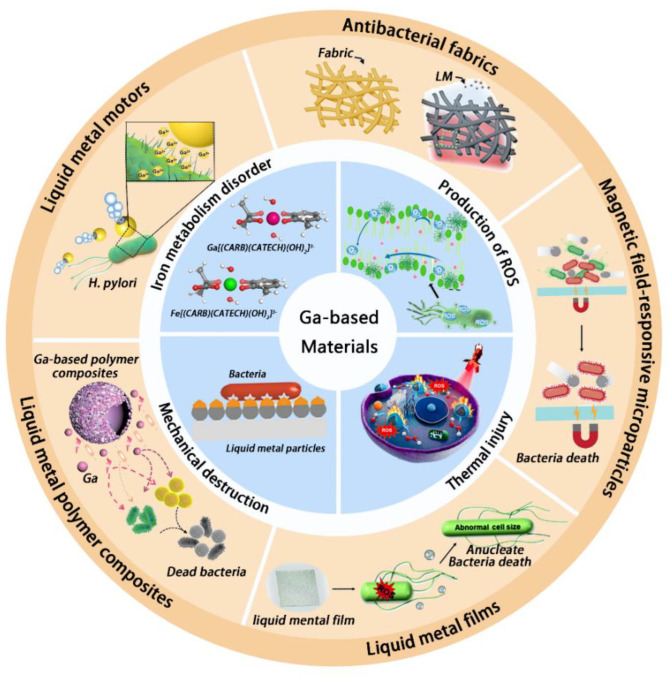
Antimicrobial mechanisms (the inner circle) and applications (the outer circle) of Ga-based liquid metal materials. The antimicrobial mechanisms include iron metabolism disorder (reprinted with permission from Ref. [32]. 2020, Elsevier, Amsterdam, The Netherlands), production of ROS (reprinted with permission from Ref. [33] 2021, KeAi Communications Co. Ltd., Beijing, China), thermal injury (reprinted with permission from Ref. [34]. 2021, Elsevier), and mechanical destruction (reprinted with permission from Ref. [35]. 2021, Wiley-VCH GmbH, Weinheim, Germany). The LM-based materials for antimicrobial applications include LM motors (reprinted with permission from Ref. [36]. 2021, Wiley-VCH GmbH), antibacterial fabrics [35], magnetic-field-responsive microparticles (reprinted with permission from Ref. [37]. 2020, American Chemical Society, Washington, DC, USA), LM films (reprinted with permission from Ref. [38]. 2021, Royal Society of Chemistry, London, UK), and LM polymer composites (reprinted with permission from Ref. [39]. 2020, Wiley-VCH GmbH).

Endowed with these intriguing properties of LM, this review is dedicated to elaborating the antibacterial mechanism of LM and mainly focuses on Ga and its alloys, including the traditional viewpoint relevant to the Fe^3+^ metabolism and several emerging opinions, as well as to summarizing the applications of LM in the field of biologically antibacterial research, such as LM motors, antibacterial fabrics, magnetic-field-responsive microparticles, LM films, and LM polymer composites (Figure 1). Last but not least, we provide an outlook on the future innovations of LM-based materials for antimicrobial activity in biomedical field, while presenting potential challenges and limitations, with the aim of achieving more practical applications in the antibacterial field.

## 2. Antimicrobial Mechanisms of LM-Based Materials

The generally accepted theory about the antibacterial mechanism of Ga is the ion competition theory [32]. Previous research has already confirmed the reliability of this theory [40,41,42]. Besides that, scientists have gradually discovered and revealed the unique bactericidal mechanism of LM, which is distinguished from the classical ion-competition mechanism. Specifically, LM micro/nanoparticles and their composites, rather than Ga^3+^ or Ga compounds, play important roles in these emerging antimicrobial mechanisms. Antibacterial agents based on metal nanoparticles have gradually become a hot research topic in recent years. Gold, silver, and copper nanoparticles have been used as antimicrobial agents [43,44,45,46]. This research also provides guidance and reference for the synthesis and application of Ga-based metal particles as antimicrobial agents [47,48,49].

### 2.1. Iron Metabolism Disorder

Metal ions are in very low amounts in organisms, while they play irreplaceable roles in biological metabolism [25]. It is necessary for certain proteins to bind with the correct metal ions to fulfill their functions. For instance, hemoglobin and myoglobin are intimately associated with the functioning of Fe^3+^ [50]. Some “alien” metal cations that are unable to be recognized and eliminated by a biological system can enter the organism and compete for protein-binding sites, thus disrupting physiological metabolism. Interestingly, introduction of a foreign metal ion for competition was adopted by scientists for disease therapies such as mental diseases [51], osteoporosis [52], cancer therapy [53,54], and anti-inflammatory therapy [26,55,56].

**Figure 2:**
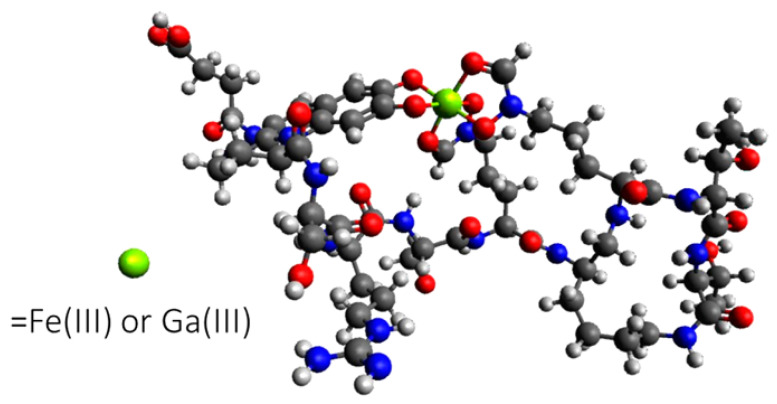
Schematic diagram of Ga^3+^ competing with Fe^3+^ for binding to pyoverdine, a siderophore synthesized by *P. aeruginosa*. Reprinted with permission from Ref. [57]. 2019, American Chemical Society.

With increasing research on the antimicrobial effect, the most widely recognized mechanism of Ga^3+^ involves the competitive activity with Fe^3+^, to bind with bacterial siderophores (as shown in Figure 2) [57]. Based on this mechanism, a series of Ga-based antibacterial drugs have been produced, based on the triggering of ion metabolic disorders such as Ga nitrate [42], Ga(III)protoporphyrin IX [58], and gallium maltolate [59].

Ga^3+^ and Fe^3+^ have many similarities in terms of structural features and chemical properties [60]. For instance, they share the same oxidation state, and both have similar coordination patterns (preferentially forming octahedral complexes rather than tetrahedral complexes) [61]. Moreover, Ga^3+^ and Fe^3+^ have similar ionization potential and electron affinity [25]. The similarities between the two, as described above, build the competitive basis for protein-binding sites [32,62]. Nevertheless, there are certain differences between Ga and Fe as well. Fe^3+^ exists in divalent and trivalent states and can undergo redox reactions for electron transfer, while Ga^3+^ cannot participate in redox reactions. If Ga^3+^ binds to a Fe-dependent enzyme, it will inactivate the enzyme due to the loss of electron transfer [25]. As a result, the normal Fe metabolism of bacteria is further disturbed.

Properties such as pH and the dielectric constant of the medium were found to have a major influence on the metal competition process [32]. As shown by conventional density functional theory (DFT) calculations [25], an acidic pH promotes the binding of Ga^3+^ to protein sites, while an alkaline pH favors the binding of Fe^3+^ to protein sites [60]. This demonstrated that the antibacterial effect of Ga^3+^ could be effectively controlled by changing the pH of the environment and, thus, reversing the metal selectivity of the Fe carrier [63].

Apart from the properties and structures of the two metals, the dielectric constant and composition of the protein-binding sites also exert an important influence on the competitive behaviors [64]. The dielectric constant of the encapsulated protein binding site is usually low. Fe^3+^ is more likely to bind to the sites with lower dielectric constants and is less likely to be interfered with by Ga^3+^ [65]. For the exposed protein-binding sites, both Fe^3+^ and Ga^3+^ can bind to them easily.

### 2.2. Production of Reactive Oxygen Species

**Figure 3:**
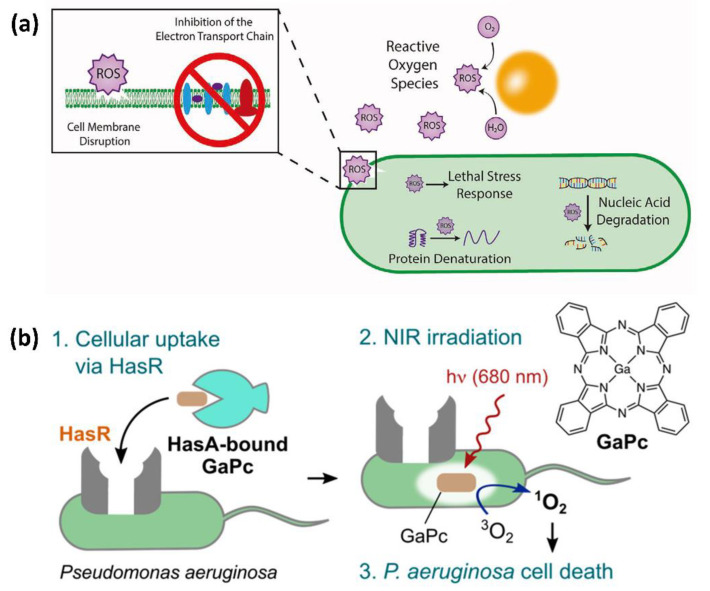
Production of reactive oxygen species (ROS) for bacterial destruction. (**a**) Damage caused by ROS in bacteria. Reprinted with permission from Ref. [71]. 2020, Wiley-VCH Verlag GmbH & Co. KGaA, Weiheim. (**b**) The process of photosterilization of bacteria with heme collection system. Reprinted with permission from Ref. [74]. 2019, American Chemical Society.

ROS, including the free and non-free radicals, refer to the single electron reduction product of oxygen [66,67]. Due to the unpaired electrons, in vivo ROS have high reactivity, which are even able to attract DNA and cause cell apoptosis [68,69]. Based on this characteristic, recent researchers found that ROS could participate in the antimicrobial activity of organism as well. As a phagocytic weapon, ROS can defend against invading pathogens [66]. The autoxidation process of NADPH enzymes produces large amounts of hydrogen peroxide as well as superoxide, and these oxidants, which react with Fe^2+^ to produce free radicals, can cause metabolic imbalance [70]. Figure 3a summarizes the damage caused by ROS to antibacterial cells [71]. Furthermore, it is important to note that the damage caused by ROS to bacteria usually involves the synergistic effects of the antimicrobial agents, such as physical damage or the enhancement of the expression of sentinel genes, etc. [72].

Under near-infrared (NIR) irradiation, photosensitive biomaterials are able to catalyze oxidative chain reactions that can produce large amounts of ROS [73]. Ga can interfere with the bacterial antioxidant enzymes in Fe metabolism and increase the sensitivity of bacteria to oxidative reactions [70]. Aimed at this principle, some research ingeniously combines Ga-based LM with photosensitizers in a synergistic manner. As is known, indocyanine green (ICG) can produce ROS under NIR laser irradiation. Combined with ICG, a Ga-based nanoparticle reagent can produce ROS by photothermal performance and provide synergistic therapeutic effects [33]. Furthermore, oxygen-deficient hollow TiO_2−x_ nanoshells (H-TiO_2−x_ NSs) can be used as a ROS generator, to share the antibacterial responsibility with Ga [39]. Likewise, investigators used the pirated heme transport system of bacteria Pseudomonas aeruginosa (*P. aeruginosa*) as well as its multidrug resistant strains to transport Ga phthalocyanine (GaPc) to the interior of target bacteria, which has promising therapeutic potential to generate ROS and sterilize the targets under NIR light (Figure 3b). Experiments demonstrated that the sterilization effect of this maneuver was more than 99.99% [74]. Morales-de-Echegaray et al. used light to transport Ga-hemoglobin, piggybacked on Ag nanoparticles, to its destination. In this case, Ga-hemoglobin is a complex of GaPppIX (a strong photosensitive compound) encapsulated in hemoglobin. The free radicals generated by the compound at the target facilitate the killing of the bacterial cells [70].

In the work of Rao et al. [38], the antibacterial mechanism of LM relying on ROS was verified. In their study, 2′-7′-dichlorofluorescein diacetate and L-ascorbic acid, which were used to mark and inhibit the production of ROS, respectively, were added to EGaln film. The results showed that the activity of bacteria increased sharply after the inhibition of ROS, indicating that the production of ROS is important factors for antibacterial performance of LM.

### 2.3. Thermal Injury

LM is able to produce heat under two main modalities: magnetic-field-induced hyperthermia [75,76] and laser-induced hyperthermia [77,78]. Compared with rigid nanoparticles for thermal therapies, the advantages of LM-based materials can realize transformation and conformable therapy, which is beneficial for blood vessel penetration and tumor coverage [31]. In current research, production of heat by LM is often applied to the elimination of cancer cells [29,34]. Based on the same mechanism, researchers have also applied LMs for the elimination of harmful microorganisms [79].

Magnetic hyperthermia relies on the ability of materials in the alternating magnetic field to generate heat for localized treatment. LM owns excellent conductivity in electricity, thus inducing a high eddy current under magnetic fields, which was recently adopted to ablate tumors [80]. In the latest study by Sun et al. [81], an implantable electrode with excellent biocompatibility and biosafety was fabricated by magnetic LM. The electrode achieved growth inhibition for tumor cells. This research results bring inspiration to the application of magnetic LM for antimicrobial applications inside the tissues.

**Figure 4:**
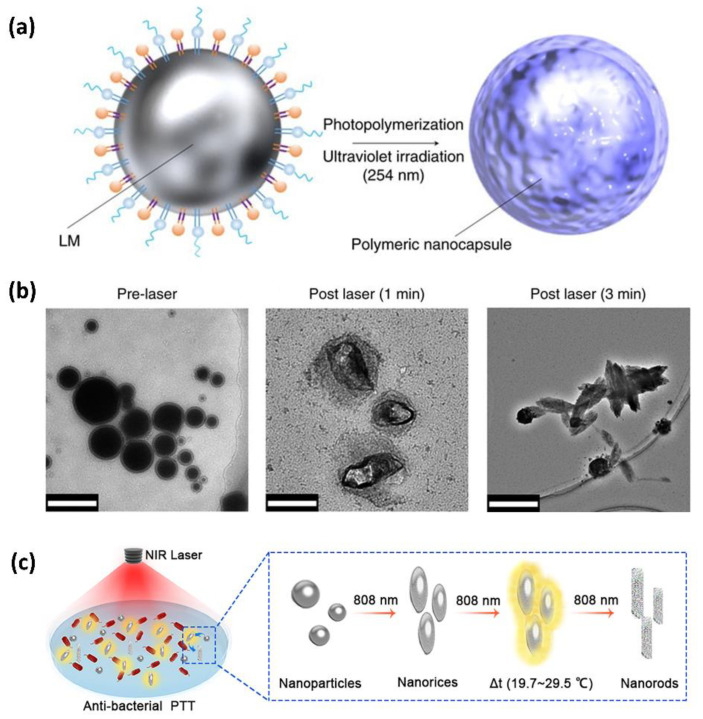
Thermal ablation of LM nanocapsule. (**a**) Schematic diagram of LM nanocapsule. (**b**) SEM images of LM nanocapsules before and after laser irradiation. Reprinted with permission from Ref. [82]. 2017, Springer Nature. (**c**) Schematic diagram of LM nanobots for antibacterial photothermal therapy. Reprinted with permission from Ref. [79]. 2020, American Chemical Society.

LM displays great potential in application of laser ablation [29,34]. Svetlana et al. [82] prepared a modified EGaIn by functionalized phospholipid photopolymerization; this LM displayed favorable hydrodispersibility and possessed a unique nanoscale capsule structure (as shown in Figure 4a). The LM nanocapsule could deform its shape with nanostructure disruption under NIR laser irradiation, and the loaded drug was, thus, released (Figure 4b). Taking advantage of this characteristic, targeted drug release as well as the elimination of tumor cells can be simultaneously achieved. Meanwhile, it was found in the experiment that the temperature on the surface of LM droplets could increase by 23 °C immediately after 5 s of irradiation and remain at this temperature for 5 min. It proves that LM nanocapsules can convert light energy into heat energy in a high efficiency under the induction of NIR, thus physically disrupting the tumors. The mechanism behind this phenomenon of photothermal conversion is related to photoexcitation resonance, which can cause rapid heating up [34,82]. Notably, in the research of Xu et al. [79], a kind of transformable LM nanobot, which was endowed with antibacterial functions, was proposed. Under NIR light exposure, LM nanobots would complete synergistic antibacterial treatment through both photothermal and chemotherapeutic effects (Figure 4c).

At the meantime, it can be noted that under the irradiation of bio-neutral NIR, the morphology of LM particles will change from rounded spheres to sharp-edged cones [83]. This process is accompanied by the release of the loaded agent and the generation of ROS.

### 2.4. Mechanical Destruction

Mechanical destruction to bacteria membranes or biofilms can be fatal to bacteria [84]. There have been many studies reporting on the damage produced by metal nanomaterials against bacteria using physical damage [85,86,87,88], such as Fe (III) oxide [89], zinc oxide [90], silver [91], etc. Compared with rigid-metal nanoparticles, LM materials have highlighted advantages in this field due to their unique shape transformation, phase transition, and excellent robustness [12]. When controlled by different external environments such as temperature changes, magnetic field strength, and light intensity, the LM nanoparticles located in the organism undergo a change in shape and damage the cells. The mechanism of physical sterilization of LM consists of two main aspects: the physical destruction of the cell membrane or cell wall and the inhibition of the electron transport chain [71,88]. The cell wall serves as a barrier between the bacteria and the external environment, and it is also responsible for the essential substance exchange required for the cellular stock [92,93,94]. The biofilm, which is a protective substrate of bacteria with robust structure, is usually difficult to be destroyed by sole chemical agents [94,95,96,97]. Yet, it has been suggested that the physical disruption of the cell structure simultaneously causes disturbance of the membrane potential and generation of free radicals that are harmful to the cell and biofilm, while cytoplasmic efflux from membrane rupture can cause irreversible damage to the cell [87,98].

LM-based mechanical disruption is gaining increasing attention, which effectively avoids the development of antibiotics and is effective to almost all the microorganisms [99]. LM can realize phase transition from liquid to solid in many ways. Under freezing, the LM droplets were able to achieve fierce transformation, even stabbing into the solid ice crystals, which can exert a large force to the surroundings This overwhelming destructive force was adopted in cryoablation to enhance the tumor therapy. [100]. On the other hand, a magnetic field can drive magnetic LM to move and trigger phase change [101]. For example, Cheeseman et al. [87] investigated the destructive effects of magnetically responsive LMs on a variety of biofilms. They controlled the shape change of LMs by magnetic fields for mechanically puncture biofilms. The rupture of bacterial cell walls and the efflux of intracellular material were visualized under electron microscope. Compared with the application of magnetic-field-controlled metal nanoparticles, light-stimulated magnetic therapy was often used for surface sterilization, making it difficult to achieve antimicrobial and antibacterial effects inside tissues where light cannot reach. However, the current control area still has certain shortcomings. For example, the treatment area can only be controlled in two-dimensional space, and the control of 3D three-dimensional space still needs further development. In actuality, the drive of LM in 3D space is possible with current technology. Researchers have shown that LM materials were able to connect a 3D electrical circuit [102].

Besides the physical damage caused by the deformation of magnetically controlled LM, the disruption of the cell plasma membrane by other materials, and the subsequent delivery of Ga nanoparticles into the cell for sterilization, is also a common mechanism. Among them, graphene can cut the cytoplasmic membrane through its sharp edges, and, subsequently, the Ga^3+^ encapsulated in graphene is released to effectively play an antibacterial role [103].

**Figure 5:**
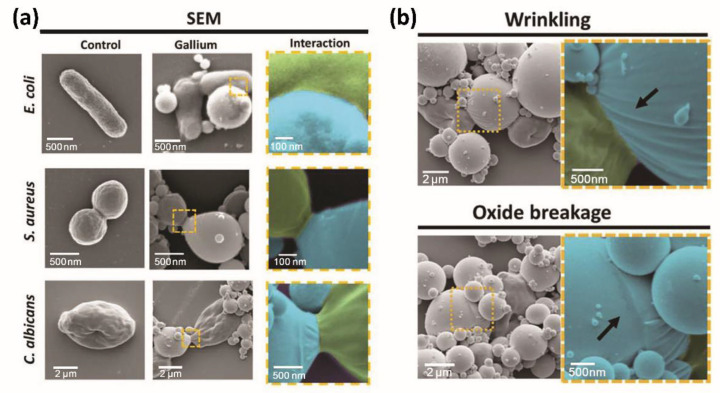
Interaction between LM and microbial cells. (**a**) SEM characterization of the interact between LM droplet (blue) and the microbial cells (green). (**b**) The formation of wrinkling and oxide breakage between LM droplet (blue) and the microbial cells (green). Reprinted with permission from Ref. [104]. 2022, Wiley-VCH GmbH.

In addition to the physical damage caused by LMNPs to bacteria, which is commonly considered at this stage, there are also researchers who have explored the biological interactions between Ga particles and cells, aiming to further illustrate and validate the antibacterial mechanism of LMNPs. In Samuel et al.’s work [104], they investigated the interactions between LM and five different cell types, including bacteria, fungi, and mammalian cells. Using super-resolution confocal laser scanning microscope (CLSM), they observed the morphological changes when cells interacted with Ga droplets in solution (Figure 5a) and found that there was folding wrinkle or even rupture of the oxide layer on the surface of the LM droplets after cell contact (Figure 5b). After analyzing this phenomenon from different perspectives, the following conclusions were tentatively obtained: this phenomenon was caused by the chemical interaction between the biomolecules on the cell surface and the LM droplets. It could also be achieved from the force test that the maximum adhesion force between the cell and LM was 0.2–0.7 nN, and the number of adhesion and detachment events increased with the contact time between Ga and the cells. This investigation provides evidence for the mechanism of interaction between LM and microbial cell, which is beneficial for Ga-based LM in biological antimicrobial applications.

## 3. Antibacterial Application of LM-Based Materials

In 1931, it was first discovered that Ga tartrate eliminated syphilis in rabbits after intramuscular or intravenous injection. This study is also the first case to prove that Ga has biological activity [105]. Then, researchers found that microbial Fe carriers, such as pyridine and deferoxamine in *P. aeruginosa*, can bind to Ga^3+^ [106]. The strategies of using Ga^3+^ as an antibacterial agent were further developed. As research on LMs increases, more LM-based antimicrobial materials are developed for antibacterial and medical therapies. This section part will summarize the typical applications of LM-based antimicrobial materials in the following areas, including LM motors, antibacterial fabrics, magnetic-field-responsive microparticles, LM films, and LM polymer composites.

### 3.1. LM Motors

Nanomotors have great potential to be utilized for drug delivery, intelligent control, and surgical robots due to the excellent flexibility and precise operation in small-scaled living organisms, such as cells, tissues, and microorganisms [107,108]. There exist some nanomotors driven by adenosine triphosphate (ATP), such as Au-Ag-Ni nanoswimmer and Pt-coated TiO_2_ [109,110].

**Figure 6:**
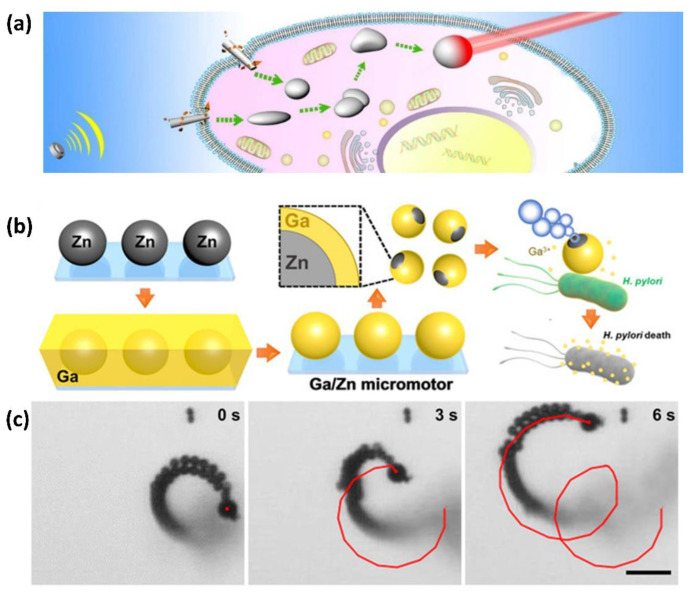
LM motors for antibacterial application. (**a**) Scheme of the intracellular transformation, fusion, and photothermal therapy of LM nanomotor. Reprinted with permission from Ref. [111]. 2018, American Chemical Society. (**b**) Synthesis of Janus Ga/Zn micromotors. (**c**) Motion of micromotors in acidic environments. Reprinted with permission from Ref. [36]. 2021, Wiley-VCH GmbH.

LM has magnetically responsive properties and favorable conductive properties. Therefore, LM-based nanomotors can respond to manipulations of external fields to achieve self-driving. For example, a nanoscale LM motor was prepared by Wang et al. [111], which consists of a liquid Ga core and a solid Ga oxide shell. Upon exposure to an ultrasound field, the nanomotor was able to move autonomously and find the target cell. The LM nanomotors could transform into a droplet while killing the cells under NIR irradiation (Figure 6a). In addition to the acoustically propelled LM motors, magnetic and electric fields can also be utilized to drive the peristaltic movement of a LM robot [112]. In the research of Li et al., they obtained functional LM, which can be manipulated accurately and rapidly [112]. Even though this motor was not at a small scale, this study lays the foundation for the future refinement of micro/nano motors.

The antimicrobial effect of Ga^3+^ relies heavily on passive diffusion, which makes the Ga^3+^ roaming and unable to reach the therapeutic site of action, thus limiting the efficiency of bacterial inhibition. To address this problem, Lin et al. [36] developed a Janus micromotor consisting of a Zn core and Ga shell. This micromotor reacts with Zinc in an acidic environment, such as stomach acid, to produce hydrogen bubbles (Figure 6b), which provide the driving power for the micromotor movement (Figure 6c). At the same time, the moving micromotor increases the diffusion efficiency of Ga^3+^ in the gut, effectively improving the antibacterial effect. In addition, the reacted micromotor degrades in vivo and does not cause harm to the organism. This micromotor has promising applications in biomedicine. For example, binding a drug to micromotors can increase the efficiency of drug release in vivo. In addition, if magnetic nanoparticles are decorated onto the micromotor, the direction of motor motion can be controlled by a magnetic field, which in turn enables targeted drug delivery.

### 3.2. Antibacterial Fabrics

**Figure 7:**
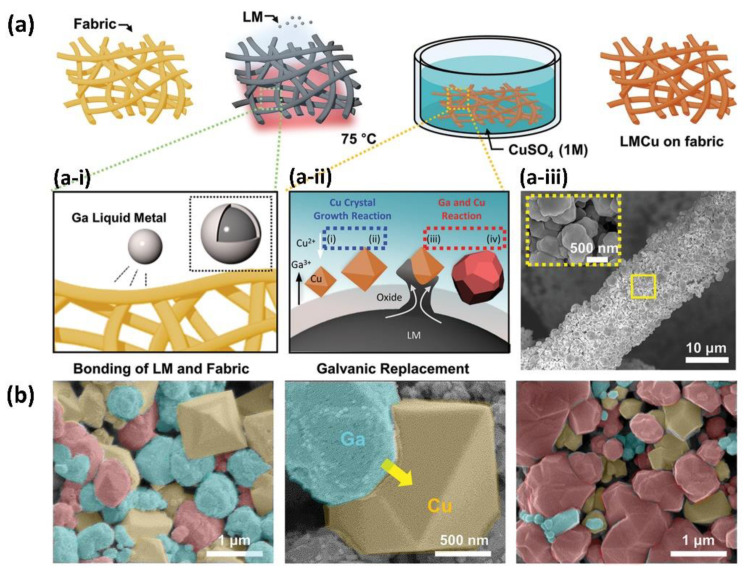
LM antimicrobial fabric. (**a**) Manufacture of antimicrobial fabrics. The bonding of LM and fabric (a-i), galvanic replacement (a-ii), and SEM images of LMCu on a single fiber (a-iii). (**b**) Surface characteristics of LM copper coating on fabric in different stages. From left to right: seed stage, growth stage, and maturity stage. Reprinted with permission from Ref. [35]. 2021, Wiley-VCH GmbH.

LM-based antimicrobial fabrics also represent new classes of antimicrobial biomaterials. As shown in Figure 7a, Kwon et al. [35] prepared a new fabric composed of LM coated with copper (LMCu). Taking advantage of the chemical properties of LM through a substitution reaction with copper ions, the fabric showed antimicrobial and antiviral effects. It is worth mentioning that the metallic coating using the galvanic replacement method has better adhesion properties (Figure 7b). To further verify the feasibility of the fabric to be used as a face mask, the researchers evaluated the adhesion of the LMCu coating under conditions of strong mechanical and airflow pressure that are higher than those of daily-life scenarios. The results showed that the coating remained firmly adhered to the fabric surface, which also demonstrates that the antimicrobial performance of the fabric can be effectively guaranteed. In this work, Ga contributes as the binder between the fabric and the copper and works as a synergistically antimicrobial agent. Notably, this work is of great importance because this antibacterial fabric can be used to fabricate masks, which can effectively reduce virus infection at this time, when COVID-19 is ravaging the world.

Fabrics for biomedical applications propose high requirements for breathability. The electrospun membranes have the advantage of high porosity. Meanwhile, the fiber structure of an electrospun membrane has a certain effect of filtering bacteria. Combining electrospun technology with LM antimicrobial material, a breathable membrane with excellent antimicrobial property can be obtained. In the research of Adrienne et al., a poly(4-hydroxybutyrate) (P4HB) electrospun membrane was prepared, which was added with GaPPIX (Ga protoporphyrin ring), thus imparting certain antibacterial properties to the membrane. The membrane was spun into a nonwoven fabric with a fiber diameter of about 700 nm. The inhibition effect on Staphylococcus aureus (S. aureus) was verified by in vitro experiments. No toxicity to human dermal fibroblasts was observed. This indicates that LM-based antimicrobial fabrics are expected to be an antimicrobial product for inhibiting skin trauma [113].

### 3.3. Magnetic-Field-Responsive Microparticles

Magnetic-field-induced antibacterial behavior mainly refers to the materials that are responsive to magnetic fields and can rupture individual microorganisms or biofilms [71]. According to different mechanisms, the application of magneto-bactericides can be divided into two categories: magnetothermal sterilization materials and magneto-controlled deformation sterilization.

**Figure 8:**
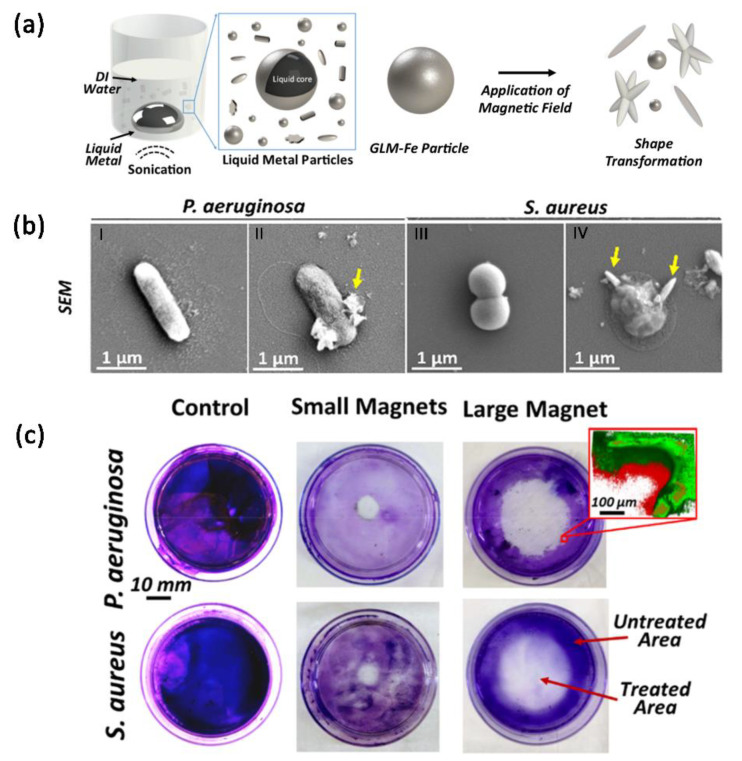
Biofilm treatment via magnetic LM antibacterial agent. (**a**) Fabrication process of magnetic Galinstan-based liquid-metal (GLM-Fe) microparticles and magnetically induced shape transformation of GLM-Fe microparticles. (**b**) SEM images of *P. aeruginosa* and S. aureus before (I, III) and after (II, IV) 90 min of magnetic-field exposure. (**c**) the spatial control of anti-biofilm activity of GLM-Fe with different magnet controls and treated biofilm, followed by staining with crystalline violet. Enlarged inset shows CLSM image of the periphery of the treated area. Reprinted with permission from Ref. [37]. 2020, American Chemical Society.

Physical damage is a much promising strategy for inhibiting biofilms. LM has a tunable mechanical property that is just suitable for this idea. Elbourne [37] et al. prepared a bactericidal material based on magnetical Galinstan-based liquid-metal microparticles (GLM-Fe). The crucial aspect of this material for bactericidal purpose is that the microparticles can transform from sphere-shaped to rod-shaped, star-shaped, and other polygon-shaped microparticles with high aspect ratios and grow sharp edges under the control of an external low-intensity rotating magnetic field (Figure 8a), thus physically destructing the bacterial biofilm as well as the pathogens (Figure 8b). The experimental results have demonstrated that the magnetically controlled GLM-Fe microparticles inactivated more than 99% of the tested Gram-negative and Gram-positive bacteria after 90 min of action.

Figure 8c illustrates the range of magnetically induced antibacterial behavior. As is shown, the size of the distinct voids increases with the magnet volume. It indicates that the treatment area can be adjusted by controlling the magnetic-field size. Most adherent biomass in the recalcitrant biofilm was also notably removed. Meanwhile, the area of sterilization also presented a better controllability with the power of the magnetic field.

Currently, LM magnetothermal sterilization materials are mostly used for cancer treatment and have yielded better results. It is promising to use this method for the treatment of bacterial cells in the future. This would be a promising method for sterilization.

### 3.4. LM Films

The synergistic bactericidal property endows LM to be applied in coatings, forming an antimicrobial film with an outstanding property. With this property, the films can be used for biomedical treatment, wound recovery, marine antifouling, etc.

**Figure 9:**
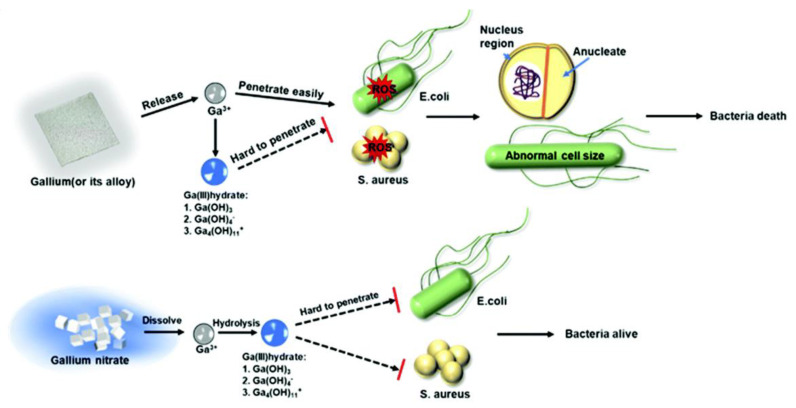
Antibacterial mechanism of eutectic gallium–indium (EGaIn) film compared to Ga nitrate. Reprinted with permission from Ref. [38]. 2021, Royal Society of Chemistry.

Li et al. [38] prepared a LM film fabricated from EGaIn alloy and verified the antimicrobial property of the film. The film was fabricated by atomized microdroplets, which were sprayed on the surface of substrates. By measuring the surface composition of the LM film and the concentration of dissolved metal ions, they proved that it is Ga, not indium, that plays a vital role in the antibacterial activity. The research confirmed the superior antibacterial properties of LM film, which could be up to a 100% antibacterial rate. They also preliminarily explored the mechanism of antibacterial activity of LM films and found that it results from the attack of ROS on bacteria, while competing with Fe^3+^ on transporter enzymes (Figure 9). Compared to conventional Ga-based agents, the direct implementation of LM nanoparticles not only overcomes bacterial resistance, but also enhances the antibacterial performance.

Besides the requirement to defeat hazardous microorganisms in biomedicine, there is currently an increasing need for antimicrobial resistance in marine decontamination. Marine fouling refers to mainly marine microorganisms (e.g., bacteria, microalgae, and mollusks) that are clinging to marine equipment [114], and the presence of these microorganisms can increase the resistance of equipment driving [115], accelerating damage to equipment due to corrosion, and even occasionally causing signal distortion as well as instrumentation failure. A growing number of researchers have tried to fabricate films by incorporating modified LM particles to coat matrices over the recent years, and these films tended to exhibit brilliant corrosion resistance and bacterial inhibition. For example, Zhang et al. [116] incorporated Ga-based LM nanoparticles into epoxy-based coatings, and it was experimentally demonstrated that the films formed by this material exhibited an outstanding corrosion resistance. Simultaneously, the incorporation of LM particles amended the defects of the original coating and improved the mechanical properties of the film. Additionally, by modifying the surface of LM nanoparticles, it can confer other properties besides antibacterial. Using modifiers such as sodium alginate, oleic acid, etc., LM nanodroplets with high stability can be successfully produced to further obtain high-quality nanofilms [117]. In the work of He et al. [118], they prepared a LM film possessing both antimicrobial as well as self-healing properties, which was made by mixing modified LM nanodroplets with polydimethylsiloxane (PDMS). The membrane exhibited satisfactory antimicrobial effect (over 90% removal of bacteria), while the self-healing properties produced by PDMS that were also achieved were attributed to the exposure of Ga atoms, inducing the further cross-linking of ethylene monomers in PDMS. Over all, with this approach, an enlightening strategy is offered for biomedical as well as LM–polymer composites research.

In spite of its inherent bactericidal role, LM has been proven to work as a catalyst to accelerate chemical reactions. Franco et al., [119] facilitated the oxidative auto-polymerization of natural polyphenols with Ga-based LM as a catalyst, resulting in nanofilms with high kinetic reactivity. As is shown, the LM nanofilms exhibited excellent antioxidant and antibacterial properties under acidic conditions. Among them, the PG film inhibited biofilm formation by 76% under the same conditions compared to the control film.

### 3.5. LM Polymer Composites

Combining LM with polymers or nanomaterials brings great convenience and advantage to the antibacterial applications [120]. For one thing, some polymeric materials are insoluble in water, so they can efficiently control the release of LMs in the system; for another, there are composites that exhibit certain bactericidal properties and can be combined with additional antibacterial agents to enhance the antibacterial performance in a variety of aspects [121].

Polymeric matrices can control the release of Ga^3+^. One research group [122], for instance, employed the polysaccharide substance carboxymethylcellulose as a binding and delivery agent to inhibit bacterial growth by delivering Ga, and the substance simultaneously displayed favorable biocompatibility on human dermal neonatal fibrillogenic cells. Notably, carboxymethyl cellulose is a gel-like substance, which makes this Ga-based antimicrobial material suitable for application under dry environment, thereby broadening the horizon of application of antimicrobial agents. More importantly, the gel substrate materials possess a certain antibacterial effect. For example, the decrease in nutrients and oxygen in the matrix during gelling can lead to cell destruction.

In that case, to control the release of Ga more precisely, the researchers can also pick polymers with degradable functions as substrates. For illustration, anti-biofilm biomaterials, fabricated by adding Ga-based composites to polyether polyurethane (PEU), exhibit certain inhibition (≥90% reduction) of both Gram-positive (*S. epidermidis*) and Gram-negative bacteria (*P. aeruginosa*) [123].

Valappil et.al. [124] combines Ga^3+^ with the carboxylic acid groups of carboxymethyl cellulose carbonate. However, this matrix is insoluble in water, so researchers believe that the digestion produced by *P. seudomonas aeruginosa* in the test mechanism promotes the separation and release of Ga in the matrix. Sodium alginate is a material widely used in the food industry. Ga-alginate prepared by combining Ga with sodium alginate also has good antibacterial properties and can effectively control the release of key ions [37]. There are also composites that promote the absorption of LM ions in vivo, thus enhancing the antibacterial effect. When Ga particles were coated with N-acetyl-cysteine (NAC), the antibacterial efficacy of the experimental group was remarkably enhanced compared to the sterilization group. This is explained by the fact that NAC has as a thiol-substituted derivative of the amino acid l-cysteine, which enables excellent water solubility [125].

The investigators also prepared Ga-chitosan complex (Ga (III)-CS) by in situ precipitation, which showed an increase in the inhibition of both Gram-positive and Gram-negative bacteria as the concentration of Ga^3+^ increased. Moreover, it was confirmed by cellular tests that the complex presented a non-toxic merit to both humans and mice [126]. All of these studies confirm that new antibiotic-free antibacterial biomaterials based on Ga have antibacterial potential in the biomedical field. By way of example, Kurtjak et al. [127] developed an antimicrobial nanocomposite containing Ga with bioactive hydroxyapatite nanorods. This material exhibited excellent antibacterial properties against *P. aeruginosa* and favorable biocompatibility with mammals as well. It is also the first report of the application of LM particles in composite with biomaterials.

Ga-based materials are widely used in dental and orthopedic medicine for their excellent antibacterial effects. The implantation of exotic biomaterials is an essential treatment tool in the currently commonly used orthopedic dental treatment, but a problem that cannot be ignored in this treatment strategy is the wound inflammation due to alien material [37]. Hence, addressing the problem of inflammation during foreign materials’ implantation is of great significance.

Titanium alloy is a metallic biomaterial commonly used in orthopedic treatment [1,128,129], which also frequently causes infections due to the biofilm formation on its surface. As a metallic material, Ga can be combined with titanium alloy and has the potential to inhibit the development of biofilm matrix [130]. Studies have also shown that Ga has an anti-resorptive effect on bone and can treat symptoms of hypercalcemia. Cochis et al. [129] metallurgically added Ga to titanium alloys and successfully achieved inhibition of S. aureus (>80% reduction of bacterial metabolic activity compared to the control group).

Likewise, Agostino et al. [131] took advantage of the antibacterial and osseointegration properties of Ga and fabricated mesoporous zirconia (ZrO_2_) materials with Ga coating. The results showed that the existence of Ga^3+^ dramatically reduced the metabolism of oral symbiotic Streptococcus salivarius and Aggregatibacter actinomycetemcomitans (*P. gingivalis* and *A. actinomycetemcomitans*) along with confirming the protective effect of Ga coating on cells against bacterial infestation The protective effect of Ga coating on cells against bacterial infection was also confirmed. The physiological significance of this antimicrobial dental material is relevant for dental implant treatment. In orthopedic treatment, Ga incorporation also offers an antimicrobial effect. Li et al. prepared a layer of LDH250 film, which is a layered double hydroxide (LDH) composed of Ga and Sr ions on Ti-based implants, by the hydrothermal method, which showed that the LDH250 sample was not affected by Ga^3+^, as a “Trojan horse” disrupts bacterial metabolism and exhibits a strong antibacterial ability, producing inhibition of both *E. coli* and *S. aureus* [132].

## 4. Conclusions and Discussion

Along with the rapid rise of drug resistances, LM materials offer a series of solutions to defeat them. In addition to the typical Ga^3+^-based antibacterial with Fe metabolism disorder, LM-based micro/nanoparticles gradually play an important role in disrupting pathogenic bacteria. For example, LM-based micro/nanoparticles can kill bacteria by the production of ROS mechanical, thermal injury, and mechanical disruption. LM-based materials present broad antibacterial applications in LM motors, antibacterial fabric, magnetic field-responsive microparticles, and LM films as well as LM polymer composites. It should be noted, nevertheless, that most studies on antibacterial LM micro/nanoparticles are still in their infancy. Thus, there are still some challenges for this new type of antibacterial materials.

LM exhibited promising performance and studies have shown that the minimum inhibitory concentration (MIC) of LM-based agents can reach 100 times that of traditional antimicrobial agents [59,133]. There is certain space to enhance the sterilizing effect for LM-based antimicrobial agents. For this issue, the LM motor is a good inspiration. It is possible to combine LM with other antibacterial agents or antibiotics, thus achieving targeted drug release and increasing their locally antimicrobial concentration and effect. Biocompatibility is the key to the application of LM in antimicrobial therapy. Ga has shown an effective antimicrobial potential in in vitro and in vivo animal studies, but it remains to be seen whether it will be validated in clinical trials.

In particular, researchers found that LM antimicrobial agents are not friendly to immunocompromised patients because prolonged use of Ga can lead to reduced immunity [134]. However, it is worth mentioning that one study has investigated that delivering LM nanodroplets into a macrophage is a potential way to modulate discrete signaling pathways, without interfering with Fe homeostasis from the usage of Ga^3+^ [135]. This study sheds light on the mechanism of LM nanodroplets–macrophage interactions, potentially enabling more pathways for Ga to be used for future biomedical and pharmaceutical applications. Therefore, by deeply investigating the emerging mechanisms of bacterial killing enabled by LM, it provides us with methods to tackle the metabolic disorder and immunity reduction of organisms caused by Ga^3+^.

Ga-based alloys have several advantages over pure Ga. By tailoring the composition, the melting point of Ga-based alloys can be lower than pure Ga (29.76 °C). Hence, the alloy can maintain good fluidity not only in biocompatible systems but also in ambient situations. What’s more, LM motors can be realized by combining Ga with other metals, such as Al or Zn, which effectively delivery the antimicrobial agents to the treatment sits or make it evenly distributed in the environment, thus, enhancing the antimicrobial efficiency of Ga-based agents. The eutectic alloys of Ga with other metals that have already been demonstrated to have an antimicrobial property, such as Ag or Cu, may present a more pronounced antimicrobial performance due to the potential combined antimicrobial effect. Overall, development of Ga-alloy-based antimicrobial agents is an important research direction for Ga-based antimicrobial agents in the future.

In addition to liquid Ga encapsulated inside, the ubiquitous oxide layers of Ga nanoparticles play a role in the antimicrobial activities that are also of interest. For example, the mechanical strength provided by the oxide layer plays a role in the mechanical destruction of bacteria [136]. It has been proposed that the oxide layers provide a versatile platform that can anchor graft molecules [21,137,138], such as silanes [139] and phosphates [140]. Based on this property, antimicrobial drugs or even antibiotics can be anchored to the Ga nanoparticles to achieve synergistic antimicrobial effects. In addition, the oxide layer can dissolve and release free Ga^3+^ at a specific pH environment [138,141]. Therefore, the oxide layer can be regarded as an ammunition depot for Ga-based antimicrobial agents.

## Data Availability

Not applicable.

## References

[1] Mular A., Shanzer A., Kozłowski H., Hubmann I., Misslinger M., Krzywik J., Decristoforo C., Gumienna-Kontecka E. (2021). Cyclic analogs of desferrioxamine e siderophore for 68Ga nuclear imaging: Coordination chemistry and biological activity in staphylococcus aureus. Inorg. Chem..

[2] English B.K., Gaur A.H., Finn A., Curtis N., Pollard A.J. (2010). The use and abuse of antibiotics and the development of antibiotic resistance. Hot Topics in Infection and Immunity in Children VI.

[3] Neill J.O. Antimicrobial Resistance: Tackling a Crisis for the Health and Wealth of Nations the Review on Antimicrobial Resistance Chaired. Antimicrob. Resist..

[4] Daeneke T., Khoshmanesh K., Mahmood N., de Castro I.A., Esrafilzadeh D., Barrow S.J., Dickey M.D., Kalantar-Zadeh K. (2018). Liquid metals: Fundamentals and applications in chemistry. Chem. Soc. Rev..

[5] Abbasi R., Tang J., Baharfar M., Zhang C., Allioux F., Zhang J., Tajik M., Yang J., Biazik J., Centurion F. (2022). Induction heating for the removal of liquid metal-based implant mimics: A proof-of-concept. Appl. Mater. Today.

[6] Ma Z.J., Huang Q.Y., Xu Q., Zhuang Q.N., Zhao X., Yang Y.H., Qiu H., Yang Z.L., Wang C., Chai Y. (2021). Permeable superelastic liquid-metal fibre mat enables biocompatible and monolithic stretchable electronics. Nat. Mater..

[7] Andrews J.B., Mondal K., Neumann T.V., Cardenas J.A., Wang J., Parekh D.P., Lin Y., Ballentine P., Dickey M.D., Franklin A.D. (2018). Patterned liquid metal contacts for printed carbon nanotube transistors. ACS Nano.

[8] Barbee M.H., Mondal K., Deng J.Z., Bharambe V., Neumann T.V., Adams J.J., Boechler N., Dickey M.D., Craig S.L. (2018). Mechanochromic stretchable electronics. ACS Appl. Mater. Interfaces.

[9] Khoshmanesh K., Tang S.Y., Zhu J.Y., Schaefer S., Mitchell A., Kalantar-Zadeh K., Dickey M.D. (2017). Liquid metal enabled microfluidics. Lab Chip.

[10] Dickey M.D. (2014). Emerging applications of liquid metals featuring surface oxides. ACS Appl. Mater. Interfaces.

[11] Cao L.X., Yu D.H., Xia Z.S., Wan H.Y., Liu C.K., Yin T., He Z.Z. (2020). Ferromagnetic liquid metal putty-like material with transformed shape and reconfigurable polarity. Adv. Mater..

[12] Kalantar-Zadeh K., Tang J.B., Daeneke T., O’Mullane A.P., Stewart L.A., Liu J., Majidi C., Ruoff R.S., Weiss P.S., Dickey M.D. (2019). Emergence of liquid metals in nanotechnology. ACS Nano.

[13] Sun X., Yuan B., Sheng L., Rao W., Liu J. (2020). Liquid metal enabled injectable biomedical technologies and applications. Appl. Mater. Today.

[14] Sun X., Wang X., Yuan B., Liu J. (2020). Liquid metal–enabled cybernetic electronics. Mater. Today Phys..

[15] Xie W.J., Allioux F.M., Ou J.Z., Miyako E., Tang S.Y., Kalantar-Zadeh K. (2021). Gallium-based liquid metal particles for therapeutics. Trends Biotechnol..

[16] Yan J., Lu Y., Chen G., Yang M., Gu Z. (2018). Advances in liquid metals for biomedical applications. Chem. Soc. Rev..

[17] Chitambar C.R. (2018). Gallium Complexes as Anticancer Drugs. Met. Ions Life Sci..

[18] Ha S.S., Xavierselvan M., Gokalp S., Labadini D., Barros S., Duong J., Foster M., Mallidi S. (2022). Eutectic gallium-indium nanoparticles for photodynamic therapy of pancreatic cancer. ACS Appl. Nano Mater..

[19] Olakanmi O., Kesavalu B., Pasula R., Abdalla M.Y., Schlesinger L.S., Britigan B.E. (2013). Gallium nitrate is efficacious in murine models of tuberculosis and inhibits key bacterial Fe-dependent enzymes. Antimicrob. Agents Chemother..

[20] Olakanmi O., Gunn J.S., Su S., Soni S., Hassett D.J., Britigan B.E. (2010). Gallium disrupts iron uptake by intracellular and extracellular francisella strains and exhibits therapeutic efficacy in a murine pulmonary infection model. Antimicrob. Agents Chemother..

[21] Wang L., Lai R., Zhang L., Zeng M., Fu L. (2022). Emerging liquid metal biomaterials: From design to application. Adv. Mater..

[22] Auger C., Lemire J., Appanna V., Appanna V.D., Kretsinger R.H., Uversky V.N., Permyakov E.A. (2013). Gallium in bacteria, metabolic and medical implications. Encyclopedia of Metalloproteins.

[23] Bernstein L.R., Kretsinger R.H., Uversky V.N., Permyakov E.A. (2013). Gallium, therapeutic effects. Encyclopedia of Metalloproteins.

[24] Choi S., Britigan B.E., Moran D.M., Narayanasamy P. (2017). Gallium nanoparticles facilitate phagosome maturation and inhibit growth of virulent mycobacterium tuberculosis in macrophages. PLoS ONE.

[25] Chitambar C.R. (2016). Gallium and its competing roles with iron in biological systems. Biochim. Biophys. Acta (BBA) Mol. Cell Res..

[26] Choi S.R., Britigan B.E., Narayanasamy P. (2019). Iron/heme metabolism-targeted gallium(III) nanoparticles are active against extracellular and intracellular *Pseudomonas aeruginosa* and acinetobacter baumannii. Antimicrob. Agents Chemother..

[27] Michalska K., Rychłowski M., Krupińska M., Szewczyk G., Sarna T., Nakonieczna J. (2022). Gallium mesoporphyrin IX-mediated photodestruction: A pharmacological trojan horse strategy to eliminate multidrug-resistant staphylococcus aureus. Mol. Pharm..

[28] Foster B.J., Clagettcarr K., Hoth D., Leylandjones B. (1986). Gallium nitrate—The 2nd metal with clinical activity. Cancer Treat. Rep..

[29] Xia N., Li N., Rao W., Yu J., Wu Q., Tan L., Li H., Gou L., Liang P., Li L. (2019). Multifunctional and flexible ZrO_2_-coated EGain nanoparticles for photothermal therapy. Nanoscale.

[30] Hu Y.Y., Chi C.W., Wang S.H., Wang L.X., Liang P., Liu F.Y., Shang W.T., Wang W.W., Zhang F.R., Li S. (2017). A comparative study of clinical intervention and interventional photothermal therapy for pancreatic cancer. Adv. Mater..

[31] Qi Y., Yu Z., Hu K., Wang D., Zhou T., Rao W. (2022). Rigid metal/liquid metal nanoparticles: Synthesis and application for locally ablative therapy. Nanomed. Nanotechnol. Biol. Med..

[32] Kircheva N., Dudev T. (2021). Competition between abiogenic and biogenic metal cations in biological systems: Mechanisms of gallium’s anticancer and antibacterial effect. J. Inorg. Biochem..

[33] Xie T., Qi Y., Li Y., Zhang F., Li W., Zhong D., Tang Z., Zhou M. (2021). Ultrasmall Ga-ICG nanoparticles based gallium ion/photodynamic synergistic therapy to eradicate biofilms and against drug-resistant bacterial liver abscess. Bioact. Mater..

[34] Yang N.L., Gong F., Zhou Y.K., Hao Y., Dong Z.L., Lei H.L., Zhong L.P., Yang X.Y., Wang X.W., Zhao Y.X. (2021). A general in-situ reduction method to prepare core-shell liquid-metal/metal nanoparticles for photothermally enhanced catalytic cancer therapy. Biomaterials.

[35] Kwon K.Y., Cheeseman S., Frias-De-Diego A., Hong H., Yang J., Jung W., Yin H., Murdoch B.J., Scholle F., Crook N. (2021). A liquid metal mediated metallic coating for antimicrobial and antiviral fabrics. Adv. Mater..

[36] Lin Z.H., Gao C.Y., Wang D.L., He Q. (2021). Bubble-propelled janus gallium/zinc micromotors for the active treatment of bacterial infections. Angew. Chem. Int. Ed..

[37] Elbourne A., Cheeseman S., Atkin P., Truong N.P., Syed N., Zavabeti A., Mohiuddin M., Esrafilzadeh D., Cozzolino D., Mcconville C.F. (2020). Antibacterial liquid metals: Biofilm treatment via magnetic activation. ACS Nano.

[38] Li L., Chang H., Yong N., Li M.X., Hou Y., Rao W. (2021). Superior antibacterial activity of gallium based liquid metals due to ga3+ induced intracellular ros generation. J. Mater. Chem. B.

[39] Yang J.J., Wang C., Liu X.L., Yin Y., Ma Y.H., Gao Y.F., Wang Y.Z., Lu Z.D., Song Y.J. (2020). Gallium-carbenicillin framework coated defect-rich hollow TiO_2_ as a photocatalyzed oxidative stress amplifier against complex infections. Adv. Funct. Mater..

[40] Centola G., Xue F., Wilks A. (2020). Metallotherapeutics development in the age of iron-clad bacteria. Metallomics.

[41] Choi S., Switzer B., Britigan B.E., Narayanasamy P. (2020). Gallium porphyrin and gallium nitrate synergistically inhibit mycobacterial species by targeting different aspects of iron/heme metabolism. ACS Infect. Dis..

[42] Choi S., Britigan B.E., Narayanasamy P. (2019). Dual inhibition of klebsiella pneumoniae and *Pseudomonas aeruginosa* iron metabolism using gallium porphyrin and gallium nitrate. ACS Infect. Dis..

[43] Ocsoy I., Paret M.L., Ocsoy M.A., Kunwar S., Chen T., You M., Tan W. (2013). Nanotechnology in plant disease management: Dna-directed silver nanoparticles on graphene oxide as an antibacterial against *Xanthomonas perforans*. ACS Nano.

[44] Turek D., Simaeys D.V., Johnson J., Ocsoy I., Tan W. (2013). Molecular recognition of live methicillin-resistant staphylococcus aureus cells using DNA aptamers. World J. Transl. Med..

[45] Ungor D., Barbasz A., Czyżowska A., Csapó E., Oćwieja M. (2021). Cytotoxicity studies of protein-stabilized fluorescent gold nanoclusters on human lymphocytes. Colloids Surf. B Biointerfaces.

[46] Czyżowska A., Barbasz A., Szyk-Warszyńska L., Oćwieja M., Csapó E., Ungor D. (2021). The surface-dependent biological effect of protein-gold nanoclusters on human immune system mimetic cells. Colloids Surf. A Physicochem. Eng. Asp..

[47] Akhtar M.S., Panwar J., Yun Y. (2013). Biogenic synthesis of metallic nanoparticles by plant extracts. ACS Sustain. Chem. Eng..

[48] Some S., Bulut O., Biswas K., Kumar A., Roy A., Sen I.K., Mandal A., Franco O.L., İnce İ.A., Neog K. (2019). Effect of feed supplementation with biosynthesized silver nanoparticles using leaf extract of *Morus indica* L. V1 on *Bombyx mori* L. (Lepidoptera: Bombycidae). Sci. Rep..

[49] Demirbas A., Büyükbezirci K., Celik C., Kislakci E., Karaagac Z., Gokturk E., Kati A., Cimen B., Yilmaz V., Ocsoy I. (2019). Synthesis of long-term stable gold nanoparticles benefiting from red raspberry (*Rubus idaeus*), strawberry (*Fragaria ananassa*), and blackberry (*Rubus fruticosus*) extracts–gold ion complexation and investigation of reaction conditions. ACS Omega.

[50] Chitambar C.R., Zivkovic Z. (1987). Inhibition of hemoglobin production by transferrin-gallium. Blood.

[51] Gould T.D., Quiroz J.A., Singh J., Zarate C.A., Manji H.K. (2004). Emerging experimental therapeutics for bipolar disorder: Insights from the molecular and cellular actions of current mood stabilizers. Mol. Psychiatry.

[52] Pilmane M., Salma-Ancane K., Loca D., Locs J., Berzina-Cimdina L. (2017). Strontium and strontium ranelate: Historical review of some of their functions. Mat. Sci. Eng. C-Mater..

[53] Lessa J.A., Parrilha G.L., Beraldo H. (2012). Gallium complexes as new promising metallodrug candidates. Inorg. Chim. Acta.

[54] Chitambar C.R. (2012). Gallium-containing anticancer compounds. Future Med. Chem..

[55] Banin E., Lozinski A., Brady K.M., Berenshtein E., Butterfield P.W., Moshe M., Chevion M., Greenberg E.P., Banin E. (2008). The potential of desferrioxamine-gallium as an anti-pseudomonas therapeutic agent. Proc. Natl. Acad. Sci. USA.

[56] Antunes L.C.S., Imperi F., Minandri F., Visca P. (2012). In vitro and in vivo antimicrobial activities of gallium nitrate against multidrug-resistant *Acinetobacter baumannii*. Antimicrob. Agents Chemother..

[57] Nicolafrancesco C., Porcaro F., Pis I., Nappini S., Simonelli L., Marini C., Frangipani E., Visaggio D., Visca P., Mobilio S. (2019). Gallium- and iron-pyoverdine coordination compounds investigated by X-ray photoelectron spectroscopy and X-ray absorption spectroscopy. Inorg. Chem..

[58] Hijazi S., Visca P., Frangipani E. (2017). Gallium-protoporphyrin ix inhibits *Pseudomonas aeruginosa* growth by targeting cytochromes. Front. Cell. Infect. Microbiol..

[59] Piatek M., Griffith D.M., Kavanagh K. (2020). Quantitative proteomic reveals gallium maltolate induces an iron-limited stress response and reduced quorum-sensing in *Pseudomonas aeruginosa*. J. Biol. Inorg. Chem..

[60] Nikolova V., Angelova S., Markova N., Dudev T. (2016). Gallium as a therapeutic agent: A thermodynamic evaluation of the competition between Ga^3+^ and Fe^3+^ ions in metalloproteins. J. Phys. Chem. B.

[61] Shannon R.D. (1976). Revised effective ionic-radii and systematic studies of interatomic distances in halides and chalcogenides. Acta Crystallogr. Sect. A.

[62] Bonchi C., Imperi F., Minandri F., Visca P., Frangipani E. (2014). Repurposing of gallium-based drugs for antibacterial therapy. Biofactors.

[63] Noujaim A.A., Lentle B.C., Hill J.R., Terner U.K., Wong H. (1979). Role of transferrin in the uptake of gallium by tumor-cells. Int. J. Nucl. Med. Biol..

[64] Larson S.M., Grunbaumi Z., Raseyz J.S. (1981). The role of transferrins in gallium uptake. Int. J. Nucl. Med. Biol..

[65] Harris W.R., Pecoraro V.L. (1983). Thermodynamic binding constants for gallium transferrin. Biochemistry.

[66] Yang Z., Min Z.J., Yu B. (2020). Reactive oxygen species and immune regulation. Int. Rev. Immunol..

[67] Dwyer D.J., Collins J.J., Walker G.C. (2015). Unraveling the physiological complexities of antibiotic lethality. Annu. Rev. Pharmacol. Toxicol..

[68] Kumar H. (2020). Healthy immunity: It’s all about immune regulation. Int. Rev. Immunol..

[69] Hong Y.Z., Zeng J., Wang X.H., Drlica K., Zhao X.L. (2019). Post-stress bacterial cell death mediated by reactive oxygen species. Proc. Natl. Acad. Sci. USA.

[70] Morales-De-Echegaray A.V., Lin L., Sivasubramaniam B., Yermembetova A., Wang Q., Abutaleb N.S., Seleem M.N., Wei A. (2020). Antimicrobial photodynamic activity of gallium-substituted haemoglobin on silver nanoparticles. Nanoscale.

[71] Cheeseman S., Christofferson A.J., Kariuki R., Cozzolino D., Daeneke T., Crawford R.J., Truong V.K., Chapman J., Elbourne A. (2020). Antimicrobial metal nanomaterials: From passive to stimuli-activated applications. Adv. Sci..

[72] Dong T.G., Dong S., Catalano C., Moore R., Liang X., Mekalanos J.J. (2015). Generation of reactive oxygen species by lethal attacks from competing microbes. Proc. Natl. Acad. Sci. USA.

[73] Imlay J.A. (2013). The molecular mechanisms and physiological consequences of oxidative stress: Lessons from a model bacterium. Nat. Rev. Microbiol..

[74] Shisaka Y., Iwai Y., Yamada S., Uehara H., Tosha T., Sugimoto H., Shiro Y., Stanfield J.K., Ogawa K., Watanabe Y. (2019). Hijacking the heme acquisition system of *Pseudomonas aeruginosa* for the delivery of phthalocyanine as an antimicrobial. ACS Chem. Biol..

[75] Obaidat I.M., Issa B., Haik Y. (2015). Magnetic properties of magnetic nanoparticles for efficient hyperthermia. Nanomaterials.

[76] Dutz S., Hergt R. (2013). Magnetic nanoparticle heating and heat transfer on a microscale: Basic principles, realities and physical limitations of hyperthermia for tumour therapy. Int. J. Hyperth..

[77] Chen Z., Wang Q., Wang H., Zhang L., Song G., Song L., Hu J., Wang H., Liu J., Zhu M. (2013). Ultrathin pegylated W_18_O_49_ nanowires as a new 980 nm-laser-driven photothermal agent for efficient ablation of cancer cells in vivo. Adv. Mater..

[78] Hu J.J., Cheng Y.J., Zhang X.Z. (2018). Recent advances in nanomaterials for enhanced photothermal therapy of tumors. Nanoscale.

[79] Xu D., Hu J., Pan X., Sánchez S., Yan X., Ma X. (2021). Enzyme-powered liquid metal nanobots endowed with multiple biomedical functions. ACS Nano.

[80] Wang D., Xie W.S., Gao Q., Yan H., Zhang J.X., Lu J.S., Liaw B., Guo Z.H., Gao F., Yin L. (2019). Non-magnetic injectable implant for magnetic field-driven thermochemotherapy and dual stimuli-responsive drug delivery: Transformable liquid metal hybrid platform for cancer theranostics. Small.

[81] Sun X.Y., Guo R., Yuan B., Wang H.Z., Duan M.H., Yang Y.X., Zhu X.Y., Wang X.L., Chen S., Cheng J.S. (2022). Stiffness tunable implanted electrode enabled by magnetic liquid metal for wireless hyperthermia. Appl. Mater. Today.

[82] Chechetka S.A., Yu Y., Zhen X., Pramanik M., Pu K., Miyako E. (2017). Light-driven liquid metal nanotransformers for biomedical theranostics. Nat. Commun..

[83] Qi Y.Q., Jin T., Yuan K., You J.Y., Shen C., Xie K.Y. (2022). Chemically stable polypyrrole-modified liquid metal nanoparticles with the promising photothermal conversion capability. J. Mater. Sci. Technol..

[84] Blair J.M.A., Webber M.A., Baylay A.J., Ogbolu D.O., Piddock L.J.V. (2015). Molecular mechanisms of antibiotic resistance. Nat. Rev. Microbiol..

[85] Li J., Nickel R., Wu J., Lin F., van Lierop J., Liu S. (2019). A new tool to attack biofilms: Driving magnetic iron-oxide nanoparticles to disrupt the matrix. Nanoscale.

[86] Mao H., Zhang B., Nie Y., Tang X., Yang S., Zhou S. (2021). Enhanced antibacterial activity of V-doped ZnO@SiO_2_ composites. Appl. Surf. Sci..

[87] Cheeseman S., Elbourne A., Kariuki R., Ramarao A.V., Zavabeti A., Syed N., Christofferson A.J., Kwon K.Y., Jung W., Dickey M.D. (2020). Broad-spectrum treatment of bacterial biofilms using magneto-responsive liquid metal particles. J. Mater. Chem. B.

[88] Foroozandeh P., Aziz A.A. (2018). Insight into cellular uptake and intracellular trafficking of nanoparticles. Nanoscale Res. Lett..

[89] Anghel I., Grumezescu A.M., Holban A.M., Ficai A., Anghel A.G., Chifiriuc M.C. (2013). Biohybrid nanostructured iron oxide nanoparticles and satureja hortensis to prevent fungal biofilm development. Int. J. Mol. Sci..

[90] Zhang L., Jiang Y., Ding Y., Povey M., York D. (2007). Investigation into the antibacterial behaviour of suspensions of ZnO nanoparticles (ZnO nanofluids). J. Nanopart. Res..

[91] Chernousova S., Epple M. (2013). Silver as antibacterial agent: Ion, nanoparticle, and metal. Angew. Chem. Int. Ed..

[92] Ruiz-Herrera J., Sentandreu R. (1989). Fungal cell wall synthesis and assembly. Curr. Top. Med. Mycol..

[93] Perez P., Ribas J.C. (2004). Cell wall analysis. Methods.

[94] Verbancic J., Lunn J.E., Stitt M., Persson S. (2018). Carbon supply and the regulation of cell wall synthesis. Mol. Plant.

[95] Donlan R.M. (2001). Biofilms and device-associated infections. Emerg. Infect. Dis..

[96] Bjarnsholt T. (2013). The role of bacterial biofilms in chronic infections. APMIS.

[97] Wu H., Moser C., Wang H.Z., Hoiby N., Song Z.J. (2015). Strategies for combating bacterial biofilm infections. Int. J. Oral Sci..

[98] Foster T.J., Geoghegan J.A., Ganesh V.K., Hook M. (2014). Adhesion, invasion and evasion: The many functions of the surface proteins of staphylococcus aureus. Nat. Rev. Microbiol..

[99] Gu H., Lee S.W., Carnicelli J., Zhang T., Ren D. (2020). Magnetically driven active topography for long-term biofilm control. Nat. Commun..

[100] Sun X., Guo R., Yuan B., Chen S., Wang H., Dou M., Liu J., He Z. (2020). Low-temperature triggered shape transformation of liquid metal microdroplets. ACS Appl. Mater. Interfaces.

[101] Sun X., Cui B., Yuan B., Wang X., Fan L., Yu D., He Z., Sheng L., Liu J., Lu J. (2020). Liquid metal microparticles phase change medicated mechanical destruction for enhanced tumor cryoablation and dual-mode imaging. Adv. Funct. Mater..

[102] Hu L., Wang H., Wang X., Liu X., Guo J., Liu J. (2019). Magnetic liquid metals manipulated in the three-dimensional free space. ACS Appl. Mater. Interfaces.

[103] Slate A.J., Karaky N., Crowther G.S., Butler J.A., Banks C.E., Mcbain A.J., Whitehead K.A. (2021). Graphene matrices as carriers for metal ions against antibiotic susceptible and resistant bacterial pathogens. Coatings.

[104] Cheeseman S., Elbourne A., Gangadoo S., Shaw Z.L., Bryant S.J., Syed N., Dickey M.D., Higgins M.J., Vasilev K., Mcconville C.F. (2022). Interactions between liquid metal droplets and bacterial, fungal, and mammalian cells. Adv. Mater. Interfaces.

[105] Levaditi C., Bardet J., Tchakirian A., Vaisman A. (1931). Le gallium, propriétés thérapeutiques dans la syphilis et les trypanosomiases expérimentales. CR Hebd Seances Acad. Sci. Ser. D Sci. Nat..

[106] Stojiljkovic I., Kumar V., Srinivasan N. (2010). Non-iron metalloporphyrins: Potent antibacterial compounds that exploit haem/HB uptake systems of pathogenic bacteria. Mol. Microbiol..

[107] Ahmed S., Wang W., Bai L.J., Gentekos D.T., Hoyos M., Mallouk T.E. (2016). Density and shape effects in the acoustic propulsion of bimetallic nanorod motors. ACS Nano.

[108] Ai S.F., Lu G., He Q., Li J.B. (2003). Highly flexible polyelectrolyte nanotubes. J. Am. Chem. Soc..

[109] Kim K., Guo J., Xu X., Fan D.L. (2015). Recent progress on man-made inorganic nanomachines. Small.

[110] Gao W., Dong R., Thamphiwatana S., Li J., Gao W., Zhang L., Wang J. (2014). Artificial micromotors in the mouse’s stomach: A step toward in vivo use of synthetic motors. ACS Nano.

[111] Wang D., Gao C., Wei W., Sun M., Guo B. (2018). Suid metal nanomachine. ACS Nano.

[112] Li F., Kuang S., Li X., Shu J., Li W., Tang S., Zhang S. (2019). Magnetically- and electrically-controllable functional liquid metal droplets. Adv. Mater. Technol..

[113] Müller A., Fessele C., Zuber F., Rottmar M., Maniura-Weber K., Ren Q., Guex A.G. (2021). Gallium complex-functionalized p4hb fibers: A trojan horse to fight bacterial infection. ACS Appl. Bio Mater..

[114] Maan A.M.C., Hofman A.H., Vos W.M., Kamperman M. (2020). Recent developments and practical feasibility of polymer-based antifouling coatings. Adv. Funct. Mater..

[115] Selim M.S., El-Safty S.A., Shenashen M.A., Higazy S.A., Elmarakbi A. (2020). Progress in biomimetic leverages for marine antifouling using nanocomposite coatings. J. Mater. Chem. B.

[116] Zhang Y., Li Z., Ye Q., Yu Q., Pei X., Yu B., Zhou F. (2019). Novel anticorrosion property of organic coating based on liquid metal. Adv. Mater. Interfaces.

[117] Hohman J.N., Kim M., Wadsworth G.A., Bednar H.R., Jiang J., Lethai M.A., Weiss P.S. (2011). Directing substrate morphology via self-assembly: Ligand-mediated scission of gallium-indium microspheres to the nanoscale. Nano Lett..

[118] He B., Du Y., Wang B., Zhao X., Liu S., Ye Q., Zhou F. (2022). Self-healing polydimethylsiloxane antifouling coatings based on zwitterionic polyethylenimine-functionalized gallium nanodroplets. Chem. Eng. J..

[119] Centurion F., Namivandi-Zangeneh R., Flores N., Tajik M., Merhebi S., Abbasi R., Mayyas M., Allioux F.M., Tang J.B., Donald W.A. (2021). Liquid metal-triggered assembly of phenolic nanocoatings with antioxidant and antibacterial properties. ACS Appl. Nano Mater..

[120] Halwani M., Yebio B., Suntres Z.E., Alipour M., Azghani A.O., Omri A. (2008). Co-encapsulation of gallium with gentamicin in liposomes enhances antimicrobial activity of gentamicin against *Pseudomonas aeruginosa*. J. Antimicrob. Chemother..

[121] Pajor K., Pajchel L., Zgadzaj A., Piotrowska U., Kolmas J. (2020). Modifications of hydroxyapatite by gallium and silver ions-physicochemical characterization, cytotoxicity and antibacterial evaluation. Int. J. Mol. Sci..

[122] Best M.G., Cunha-Reis C., Ganin A.Y., Sousa A., Johnston J., Oliveira A.L., Smith D.G.E., Yiu H.H.P., Cooper I.R. (2020). Antimicrobial properties of gallium(III)- and iron(III)-loaded polysaccharides affecting the growth of escherichia coli, staphylococcus aureus, and *Pseudomonas aeruginosa*, in vitro. ACS Appl. Bio Mater..

[123] Ma H., Darmawan E.T., Zhang M., Zhang L., Bryers J.D. (2013). Development of a poly(ether urethane) system for the controlled release of two novel anti-biofilm agents based on gallium or zinc and its efficacy to prevent bacterial biofilm formation. J. Control. Release.

[124] Valappil S.P., Yiu H.H.P., Bouffier L., Hope C.K., Evans G., Claridge J.B., Higham S.M., Rosseinsky M.J. (2013). Effect of novel antibacterial gallium-carboxymethyl cellulose on *Pseudomonas aeruginosa*. Dalton Trans..

[125] Young M., Ozcan A., Lee B., Maxwell T., Andl T., Rajasekaran P., Beazley M.J., Tetard L., Santra S. (2019). N-acetyl cysteine coated gallium particles demonstrate high potency against *Pseudomonas aeruginosa* pao1. Pathogens.

[126] Akhtar M.A., Hadzhieva Z., Ilyas K., Ali M.S., Peukert W., Boccaccini A.R. (2021). Facile synthesis of gallium (III)-chitosan complexes as antibacterial biomaterial. Pharmaceutics.

[127] Kurtjak M., Vukomanovic M., Kramer L., Suvorov D. (2016). Biocompatible nano-gallium/hydroxyapatite nanocomposite with antimicrobial activity. J. Mater. Sci.-Mater. Med..

[128] Khosravanihaghighi A., Koshy P., Yasir M., Romanazzo S., Lovric V., Kilian K.A., Willcox M.D., Walsh W.R., Sorrell C.C. (2022). Production of antibacterial activity and bone cell proliferation by surface engineering of Ga- or Mn-doped ceria-coated biomedical titanium alloy. Adv. Eng. Mater..

[129] Cochis A., Azzimonti B., Chiesa R., Rimondini L., Gasik M. (2019). Metallurgical gallium additions to titanium alloys demonstrate a strong time-increasing antibacterial activity without any cellular toxicity. ACS Biomater. Sci. Eng..

[130] Shokri M., Kharaziha M., Tafti H.A., Eslaminejad M.B., Aghdam R.M. (2022). Synergic role of zinc and gallium doping in hydroxyapatite nanoparticles to improve osteogenesis and antibacterial activity. Biomater. Adv..

[131] Agostino A.D., Tana F., Ettorre A., Pavarini M., Serafini A., Cochis A., Scalia A.C., Rimondini L., De Giglio E., Cometa S. (2021). Mesoporous zirconia surfaces with anti-biofilm properties for dental implants. Biomed. Mater..

[132] Li K., Tian H., Guo A., Jin L., Chen W., Tao B. (2022). Gallium (Ga)–strontium (Sr) layered double hydroxide composite coating on titanium substrates for enhanced osteogenic and antibacterial abilities. J. Biomed. Mater. Res. A.

[133] Ekkelenkamp M.B., Cantón R., Díez-Aguilar M., Tunney M.M., Fluit A. (2019). Susceptibility of *Pseudomonas aeruginosa* recovered from cystic fibrosis patients to murepavadin and thirteen comparator antibiotics. Antimicrob. Agents Chemother..

[134] Li F., Liu F., Huang K., Yang S. (2022). Advancement of gallium and gallium-based compounds as antimicrobial agents. Front. Bioeng. Biotechnol..

[135] Zhang C., Yang B., Biazik J.M., Webster R.F., Xie W., Tang J., Allioux F., Abbasi R., Mousavi M., Goldys E.M. (2022). Gallium nanodroplets are anti-inflammatory without interfering with iron homeostasis. ACS Nano.

[136] Lin Y., Liu Y., Genzer J., Dickey M.D. (2017). Shape-transformable liquid metal nanoparticles in aqueous solution. Chem. Sci..

[137] Li H., Qiao R., Davis T.P., Tang S. (2020). Biomedical applications of liquid metal nanoparticles: A critical review. Biosensors.

[138] Lin Y., Genzer J., Dickey M.D. (2020). Attributes, fabrication, and applications of gallium-based liquid metal particles. Adv. Sci..

[139] Goerl U., Hunsche A., Mueller A., Koban H.G. (1997). Investigations into the silica/silane reaction system. Rubber Chem. Technol..

[140] Li M., Liu J., Xu Y., Qian G. (2016). Phosphate adsorption on metal oxides and metal hydroxides: A comparative review. Environ. Rev..

[141] Lu Y., Hu Q., Lin Y., Pacardo D.B., Wang C., Sun W., Ligler F.S., Dickey M.D., Gu Z. (2015). Transformable liquid-metal nanomedicine. Nat. Commun..

